# The transcriptomics of an experimentally evolved plant-virus interaction

**DOI:** 10.1038/srep24901

**Published:** 2016-04-26

**Authors:** Julia Hillung, Francisco García-García, Joaquín Dopazo, José M. Cuevas, Santiago F. Elena

**Affiliations:** 1 Instituto de Biología Molecular y Celular de Plantas, Consejo Superior de Investigaciones Científicas-UPV, Campus UPV CPI 8E, Ingeniero Fausto Elio s/n, 46022 València, Spain; 2 Computational Genomics Department, Centro de Investigación Príncipe Felipe (CIPF), Eduardo Primo Yúfera 3, 46012 València, Spain; 3 Bioinformatics of Rare Diseases (BIER), CIBER de Enfermedades Raras (CIBERER), 46012 València, Spain; 4 Functional Genomics Node, INB at CIPF, 46012 València, Spain; 5 The Santa Fe Institute, 1399 Hyde Park Road, Santa Fe NM 87501, USA

## Abstract

Models of plant-virus interaction assume that the ability of a virus to infect a host genotype depends on the matching between virulence and resistance genes. Recently, we evolved tobacco etch potyvirus (TEV) lineages on different ecotypes of *Arabidopsis thaliana*, and found that some ecotypes selected for specialist viruses whereas others selected for generalists. Here we sought to evaluate the transcriptomic basis of such relationships. We have characterized the transcriptomic responses of five ecotypes infected with the ancestral and evolved viruses. Genes and functional categories differentially expressed by plants infected with local TEV isolates were identified, showing heterogeneous responses among ecotypes, although significant parallelism existed among lineages evolved in the same ecotype. Although genes involved in immune responses were altered upon infection, other functional groups were also pervasively over-represented, suggesting that plant resistance genes were not the only drivers of viral adaptation. Finally, the transcriptomic consequences of infection with the generalist and specialist lineages were compared. Whilst the generalist induced very similar perturbations in the transcriptomes of the different ecotypes, the perturbations induced by the specialist were divergent. Plant defense mechanisms were activated when the infecting virus was specialist but they were down-regulated when infecting with generalist.

Emerging viruses are recognized as a threat not only to human health but also for crops, livestock and even for endangered wild species. Most RNA viruses are characterized by their high genetic variability due to replication without error proof, short generation times and very large populations^1^,^2^,^3^,^4^,^5^. It is difficult to predict and, even more, to control the amount and fate of RNA virus genetic diversity during outbreaks and seasonal epidemics, and certainly RNA viruses are of main pathological, epidemiological, ecological, and evolutionary interest. Emerging viruses are the result of the complex interaction of host pathogen dynamics and environmental factors such as the number of reservoir and potentially susceptible hosts and transmission vectors^2^,^3^,^5^,^6^,^7^. As generally accepted, host genetic diversity is a significant factor in the emergence, spread, and prevalence of infectious diseases, but it is not clear how much of such diversity is required to change the evolutionary fate of a virus^8^. Consequently, to better understand the evolution of viruses as obligate cellular parasites, it is essential to study them through a different perspective, focusing on where they thrive. Most evolution experiments performed with plant viruses to address the role of host diversity in the evolutionary fate of viral populations have focused in the use of different host species^9^,^10^,^11^,^12^,^13^,^14^,^15^,^16^,^17^,^18^,^19^. By contrast, very few studies have experimentally explored the role of within-host species diversity in resistance and susceptibility genes into the evolution of plant viral pathogens.

*Arabidopsis thaliana* L. is an excellent experimental system to study the role of intraspecific variability in the evolution of viral pathogens. *A. thaliana* ecotypes have a unique ecology and are adapted to specific environments, as opposed to differing only in genotype from other varieties^20^. Susceptibility to viral infection varies among ecotypes^21^: while some allow for long-distance movement out from the inoculated to non-inoculated leaves, other only support replication in inoculated leaves but not systemic movement. In the well studied case of *Tobacco etch virus* (TEV; genus *Potyvirus*, family *Potyviridae*), susceptibility depends on the *Restricted TEV Movement* (*RTM*) multigenic system composed at least by the *RTM1, RTM2* and *RTM3* loci^21^,^22^,^23^,^24^,^25^,^26^,^27^. The presence of dominant alleles in all three loci is necessary for resistance, homozygous recessive mutations at any of these loci result in systemic infection^24^,^25^.

A series of previous studies have explored the phenotypical and transcriptomic differences associated with the infection of *A. thaliana* ecotypes Di-2, Ei-2, L*er*-0, Oy-0, St-0, and Wt-1 by a wild-type ancestral TEV strain from tobacco and by the L*er*-0-adapted isolate TEV-*At*17b^19^,^28^,^29^,^30^,^31^,^32^. These ecotypes differed in their susceptibility to infection with TEV as well as in the severity of the symptoms induced^29^,^30^. Ei-2 and L*er*-0 had *rtm1* and St-0 had *rtm3* alleles in homozygosis and were susceptible to infection with the wild-type virus^29^; Di-2, Oy-2 and Wt-1 had no susceptibility alleles and were resistant to infection with the wild-type virus^29^. However, all ecotypes were susceptible to infection with isolate TEV-*At*17b^29^,^30^. Indeed, these ecotypes can be classified into two groups according to their response to infection with TEV-*At*17b. At the one hand, Ei-2 and Wt-1 develop milder symptoms than the other ecotypes, virus titers were variable but lower than in the other ecotypes, and genes involved in abiotic stresses and in the construction of new tissues tend to be up-regulated^30^. At the other hand Di-2, St-0 and L*er*-0 show symptoms ranging from moderate to severe, infected plants always accumulated high viral titers, and genes involved in plant defenses were significantly enriched among the up-regulated functional categories^30^. A follow up study tackled the question of whether these genetic differences on susceptibility to infection could condition the subsequent evolution of TEV-*At*17b^32^. After experimental evolution, the evolved viral strains were inoculated in their local host ecotype as well as in the four alternative ecotypes. Each one of the virus-host combination was analyzed for relative fitness, virulence and infectivity^32^. Permissive hosts selected for specialist viruses whereas restrictive hosts selected for generalist viruses. Specialist viruses we those that replicated well in a host ecotype but disproportionally worse in the alternative host ecotypes. By contrast, a generalist virus would be one replicating equally well across all host ecotypes. No evolved virus was superior to all others in every ecotype and no ecotype was resistant against all evolved viruses. Viral lineages evolved in the same ecotype have also accumulated genetic variation and diverged from each other. The genome of evolved TEV-*At*17b lineages was sequenced and cases of host-dependent convergent mutations were found^31^,^32^.

The present study aims to explore the molecular basis of the interaction between these 15 viral lineages and the ecotypes wherein they had evolved (*i.e*, local associations) as well as the interaction with the rest of ecotypes not experienced during the evolution experiment (*i.e.*, foreign associations). We will pay special attention to disentangling responses that are ecotype-specific from those that may be common among ecotypes when infected with local and foreign viral lineages. We have performed a set of transcriptomic experiments using the Agilent *Arabidopsis* (V4) gene expression microarray 4 × 44K technology. mRNA microarray assays make feasible quantifying the expression for almost all genes of *A. thaliana* during plant response triggered by virus infection. This is a comparative transcriptomics study, and as such, it builds upon a tremendous amount of information about the changes in expression of a multitude of genes and of derived biological functions. Rather than looking at endless lists of genes and functions, we will focus our attention on general questions and try to infer general changes in plant responses to infection as the virus adapts to its new hosts. The questions tackled in the followings section are (1) which classes of host genes are responding in a specific manner to the infection with local viral lineages, and thus can be seen as potential drivers for virus adaptation? (2) Do they differ among host ecotypes? (3) Upon adaptation to a given local host ecotype, does the evolved virus interact differently with its ancestral host, L*er*-0? If so, the interaction with which classes of genes has been affected? And finally, (4) are transcriptomic responses induced by specialist and generalist viruses different from each other across all five host ecotypes.

## Results and Discussion

### Analysis of local adaptation: comparisons between expression profiles of plants infected with local *vs* foreign viral lineages

The first question tackled was whether the transcriptomic profiles from plants infected with viruses locally evolved were more similar among them than from those observed for plants from the same ecotype but infected with foreign viral lineages. The statistical contrasts were of the form “expression in plants infected with evolved viruses” differ from “expression of mock-inoculated plants of the same ecotype” (see “Methods: microarray data analyses” for a precise mathematical definition of the contrasts). We quantified the similarity between all possible pairs of expression profiles using the Pearson product-moment correlation coefficient; then the coefficients computed for the three independent lineages evolved in the same local host were averaged. The results from this analysis are shown in Table 1. The diagonal in Table 1 shows the average correlation coefficients (±1 SEM; *n* = 3) for the viral lineages on their corresponding local host ecotypes. Focusing first in the diagonal elements, the correlations were significantly positive, suggesting a significant degree of evolutionary convergence among viral lineages evolved in the same host ecotype. However, the extent of convergence was variable among ecotypes: while for Di-2 and L*er*-0 the average similarity was slightly over 0.60, it was around 0.80 for lineages evolved in St-0, Ei-2 and Wt-1, suggesting that the latter lineages may impose more severe constraints to virus adaptation and thus the number of possible solutions available for the evolving viral populations are limited.

**Figure 1:**
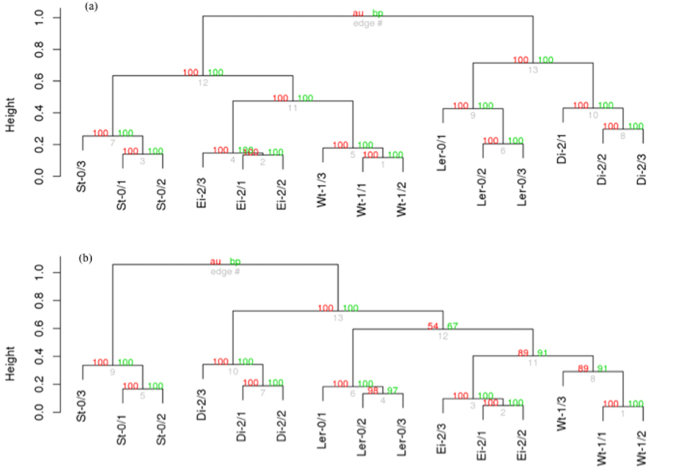
Similarity in the response of local host to their local viruses. (**a**) Dendrogram grouping transcriptomic responses to infection with each of the evolved viruses on their corresponding local hosts. (**b**) Dendrogram grouping functional profiles obtained after infecting each of the evolved viruses on their corresponding local hosts. Red numbers represent the approximately unbiased support of each cluster (percentage *P*-value) computed by multiscale bootstrapping. Green numbers represent the support of each cluster based on a standard bootstrapping. Grey numbers indicate the node label.

Next, we evaluated whether a given viral lineage interacted in a similar way with all host ecotypes. In other words, whether the evolutionary convergence observed in the patterns of gene expression of infected local ecotypes was similar for the foreign hosts. The off-diagonal numbers in Table 1 show the Pearson correlation coefficients between the average patterns of gene expression (*i.e.*, averaging the expression of each gene across the *n* = 3 independently evolved lineages) across ecotypes. Interestingly, the magnitude of the correlation coefficients was lower than discussed above for local associations and highly variable among ecotypes. As showed in Table 1 in pairs Di-2 and Wt-1 and Ei-2 and L*er*-0 the associations were very weak (*r* < 0.2), yet significant (*P* < 0.001). Association was weak for pairs L*er*-0 and Wt-1 and St-0 and Wt-1 (0.2 < *r* < 0.4) and moderate for pairs Di-2 and L*er*-0, Ei-2 and St-0 and Ei-2 and Wt-1 (0.4 < *r* < 0.6). Interestingly, expression profiles among Di-2 and Ei-2 and expression profiles among St-0 and Di-2 and L*er*-0 were weakly negatively correlated among them, suggesting that St-0-adapted viral lineages interacted with its local host in a radically different way as other lineages did. To better visualize the similarity in gene expression induced by infection with the evolved viral lineages, Fig. 1a shows a dendrogram based on the pairwise correlation coefficients. Overall, two significant groups of virus-ecotype interactions exist. In the first group, lineages evolved in L*er*-0 and Di-2 formed a cluster, which is subsequently divided into two branches, each one grouping lineages of corresponding ecotype. The second cluster incorporated lineages evolved in Ei-2, St-0 and Wt-1, though they segregated in separated ecotype-defined subgroups. Within this second cluster, Wt-1- and Ei-2-evolved lineages were more similar in their interaction with the hosts than St-0-evolved lineages.

**Figure 2:**
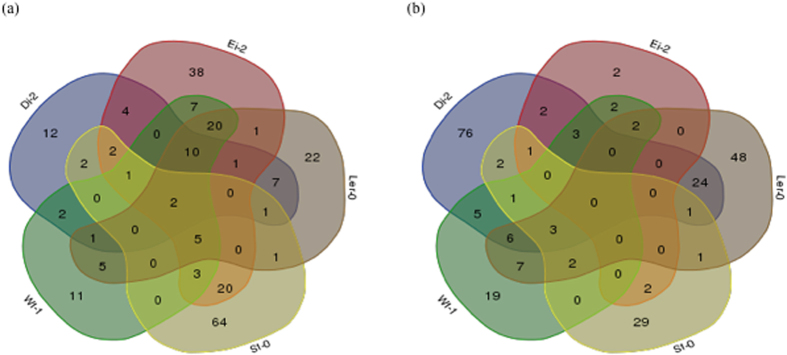
Venn Diagrams of enriched functional categories in ecotypes infected with local evolved viral lineage in contrast to corresponding mock-infected ecotypes. (**a**) For up-regulated functional categories and (**b**) for down-regulated functional categories.

Next, a gene set analysis (GSA) using the highly sensitive logistic regression method (LRpath)^33^,^34^ was used to identify functional categories of altered host genes for each viral lineage on each of the five plant ecotypes. All genes detected in each microarray slide, ranked by their differential expression level, were used for the analyses. Comparing these ranked lists of genes in a pairwise manner, we looked for sets of genes whose enrichment was different from a healthy plant, regardless the size of the effect was significant or not, in one of the two lists being compared. Then, we looked for blocks of overrepresented genes that shared functions and we could identify enriched functional categories (GO terms) for each ecotype. Furthermore, the analyses generated two levels of results: (1) all significant GO terms and (2) a group constituted only by significant more specific non-redundant GO terms. To identify consistent features among lineages and ecotypes we evaluated the intersections of non-redundant functions. Given that GO terms could have different names yet pointing to the same function, the intersection between parallel replicates should be taken just as a global picture of similarities. Even keeping this drawback in mind, it is noticeable that lineages evolved in the same ecotypes shared more functional categories than those evolved on different ecotypes. For over-expressed genes, the number of ecotype-specific enriched functional categories ranged between 11 (for Wt-1-evolved lineages) and 64 (for St-0-evolved lineages) and there were only 2 functional categories shared by all five ecotypes-evolved lineages (Fig. 2a). These categories are *response to cadmium ion* (GO:0046686) and *photorespiration* (GO:0009853). For down-regulated categories, the number of ecotype-specific enriched GO terms ranged between 2 (Ei-2-evolved lineages) and 76 (Di-2-evolved lineages) and there were no common functional categories shared by all ecotypes, but some cases between pairs of ecotypes. The larger similarity in down-regulated GO terms was for L*er*-0 and Di-2 (24), while Ei-2, Ler-0 and Wt-1 share 20 up-regulated functional categories. The same number is also shared between ecotypes Ei-2 and St-0.

To further explore the similarities and differences in terms of functional annotations, we proceeded as above but now computing Pearson correlation coefficients between the lists of enriched GO terms generated for each evolved viral lineage on each plant ecotype. Table 2 shows the average correlation coefficients. As above, the diagonal elements represent the average correlations between lineages evolved in the same ecotype (local combinations). The off-diagonal elements represent the correlations among the corresponding pairs of ecotypes averaged among lineages (foreign combinations). All local combinations show highly significant and positive correlations among independently evolved lineages, suggesting a strong convergence in terms of the biological functions altered upon infection. As in the case of gene expression profiles, foreign combinations show more variability, with pairs of ecotypes showing no similarity in their response to infection (*e.g*., St-0 and Wt-1) or even significant negative associations (Di-2 and St-0 and L*er*-0 and St-0) and others showing significant similarities (ranging from 0.136 for Ei-2 and St-0 to 0.667 for Ei-2 and Wt-1). Pairwise correlation coefficients were used to build a dendrogram that shows the similarity among viral lineages in the lists of GO terms generated on each ecotype (Fig. 1b). Viral lineages evolved in St-0 had functionally different expression pattern than the other ecotypes, thus forming a clearly independent cluster. The rest of ecotypes form a second cluster whereas no ecotypes were more similar to each other, as the cluster had a nested structure. It is noteworthy that this functional clustering was different from the clustering generated from the transcriptomic profiles, and from the clustering generated using the functional response data of plants infected with the ancestral strain TEV-*At*17b^30^.

From these set of analyses, we can conclude that the expression profiles of host’s mRNAs and their corresponding functional profiles were heterogeneous among ecotypes, although a significant degree of parallelism exist among lineages evolved in the same ecotype. Overall, two different types of transcriptomic response could be identified that are similar to the responses observed when plants were infected with the ancestral TEV-*At*17b^30^. These observations suggest that the evolution of virus-host interactions was restricted by the genetic characteristics of the host. However, clustering based on functional response shows a different topology, suggesting that similar functional categories were probably altered by the effect of viral infection on different genes in each ecotype.

### Disentangling ecotype-specific from general drivers of virus adaptation

**Figure 3:**
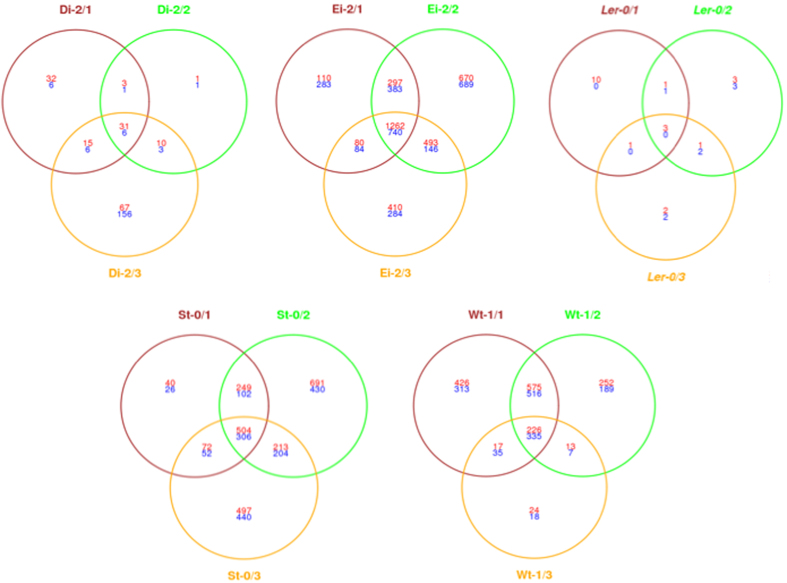
Venn diagrams of genes shared between locally adapted and ancestral viruses when infecting the corresponding local host ecotype. Each circle indicates an independent replicate of viral evolution in each given plant ecotype. Intersection areas indicate the number of common differently expressed genes between independent lineages. In red, number of over-expressed genes, in blue number of under-expressed genes.

In this second set of analyses we aimed to identify ecotype-specific and universal drivers of viral adaptation. In other words, we are comparing the response of each given ecotype to infection with the locally-adapted viral lineages with their response to the infection with the ancestral TEV-*At*17b isolate^30^. Then, the lists of host genes whose expression is different in plants infected with the ancestral and the evolved viruses were compared in search of commonalities. Both series of samples were simultaneously normalized and control values (non-infected plants) were subtracted from the corresponding samples. In this case the statistical contrasts were of the form “genes altered upon infection with the evolved virus” differ from “genes altered upon infection with the ancestral virus” for plants of the same ecotype (see “Methods: microarray data analyses” for a precise mathematical definition of the contrasts). Figure 3 shows the number of genes whose expression was significantly different between ecotypes infected with evolved and ancestral lineages. Plants infected with viral lineages evolved in ecotypes Di-2 (37 genes shared by all three lineages) and L*er*-0 (only 3 genes were shared by the three lineages) have the lowest number of differentially expressed genes compared to infection with the ancestral virus. By contrast, plants infected with the lineages evolved in Ei-2 showed the largest amount of genes with differential expression/repression (2002 genes shared by all three lineages). Plants infected with St-0- and Wt-1-evolved viral lineages had intermediate, and similar, numbers of differentially expressed genes as a consequence of viral evolution on these ecotypes (810 and 561, respectively, shared by the three replicates). Noteworthy, in all diagrams shown in Fig. 3, the largest amount of altered genes always belongs to the category of genes shared by the three independent lineages, again pointing to a host ecotype-driven outcome of the evolutionary process.

Next, enriched functional categories for each lineage were identified. To summarize the GO terms for biological processes, super clusters of functional categories were determined using the ReViGO tool^35^ and represented in Tables 3 and 4 for up- and down-regulated categories, respectively. Although all ecotypes include a core set of super categories that are specific (*i.e.*, not shared with other ecotypes), some up- and down-regulated functional super clusters were shared among two or more ecotypes (Tables 3 and 4). For instance, 98 categories were found for over-expressed genes, 74 of which were ecotype-specific (8 for Di-2, 22 for Ei-2, 12 for L*er*-0, 20 for St-0, and 12 for Wt-1). The up-regulated categories that were most commonly shared by more than one ecotype were *response to wounding* (GO:0009611; Di-2, Ei-2 and Wt-1) and *protein folding* (GO:0006457; Ei-2, St-0 and Wt-1). One of the categories shared by ecotypes Di-2 and L*er*-0 were *maltose metabolic process* (GO:0000023). Disaccharide maltose is a major photosynthetic product in *A. thaliana*^36^ and is formed from two units of glucose produced when amylase breaks down starch, the main form by which plants store carbohydrate. In addition, in this ecotypes were modified genes involved in maintenance of *cation homeostasis* (GO:0055080), *anthocyanin-containing compound biosynthetic process* (GO:0009718), that includes pathways resulting in the formation of anthocyanidins, and *metabolic process of p-aminobenzoic acid* (GO:0046482), an intermediate in the synthesis of folic acid needed for DNA and RNA amplification.

Ei-2 and Wt-1, both had over-expressed genes involved in the following processes: (1) *Ethylene biosynthetic process* (GO:0009693). The plant hormone ethylene is involved in many aspects of the plant life cycle^37^, including seed germination, root hair development, root nodulation, flower senescence, abscission, and fruit ripening. The production of ethylene is tightly regulated by internal signals during development and in response to environmental stimuli from biotic (*e.g.*, pathogen) and abiotic stresses, such as wounding, hypoxia, ozone, chilling, or freezing^38^. (2) *Vesicle-mediated transport* (GO:0016192) is a cellular process by which substances are moved in membrane-bounded vesicles; transported substances are enclosed in the vesicle lumen or located in the vesicle membrane^39^. (3) *Detection of biotic stimulus* (GO:0009595) conceals a series of processes in which a biotic stimulus, once caused or produced by a living organism, is received and converted into a molecular signal^39^. (4) *Response to cyclopentenone* (GO:0010583). Cyclopentenones are derivates from polyunsaturated fatty acids and are structurally and functionally similar to jasmonic acid, a regulator of plant responses to abiotic and biotic stresses as well as plant growth and development^40^. (5) *Defense response by callose deposition* (GO:0052542). Callose is a plant polysaccharide and can be deposited as cell-wall appositions and form an effective barriers in early stages of pathogen invasion and also serves as a matrix in which antimicrobial compounds can be deposited^41^. And (6) *aspartate family amino acid catabolic processes* (GO:0009068) are implicated in the breakdown of amino acids of the aspartate family, comprising asparagine, aspartate, lysine, methionine, and threonine.

Plants from ecotypes St-0 and Di-2 had two up regulated clusters in common: *negative regulation of catalytic activity* (GO:0043086) *and photosystem II assembly* (GO:0010207). St-0 and Wt-1 shared in common clusters such as *response to high light intensity* (GO:0009644) and *secondary metabolite biosynthetic process (*GO:0044550). Up-regulated categories of immune response as *response to hydrogen peroxide* (GO:0042542), and *jasmonic acid biosynthetic process* (GO:0009695) were significantly enriched in ecotypes St-0 and Wt-1. Alterations of an existing cellulose and pectin-containing cell wall that could result in loosening, increased extensibility or disassembly of cell wall was also up-regulated by the virus infection under GO designation *plant-type cell wall modification* (GO:0009827). *Golgi organization* (GO:0007030), a process that is carried out at the cellular level which results in the assembly, arrangement of constituent parts, or disassembly of the Golgi apparatus and *drug transmembrane transport* (GO:0006855), a process in which a drug is transported from one side of a membrane to the other by means of some agent such as a transporter or pore were up-regulated in ecotypes Di-2 and Ei-2. Both categories are closely related to viral replication and to cell-to-cell transport of virions or of replicative complexes. Plant-type *cell wall biogenesis* (GO:0009832) and *response to cadmium ion* (GO:0046686) are common categories affected in ecotypes Ei-2 y L*er*-0. Plant response to cadmium evokes a number of parallel and/or consecutive events at molecular, physiological and morphological levels. Potential effects of cadmium on host-pathogen interactions have been examined and it was demonstrated that in tobacco plants it induced the production of putative antiviral factors capable to inhibit the systemic spread of the *Turnip vein clearing virus*^42^. In experimental condition without obvious stimulus of cadmium, it is easy to think that the pathway does not exclusively respond to cadmium, but it could be activated by virus infection. *mRNA modification* (GO:0016556) is the only category that the ecotypes Ei-2 and St-0 had in common, but this process can involve a variety of different impacts in host-virus interaction, ranged between protein biosynthesis to resistance of the plant.

Among the 90 down-regulated responses, 72 were ecotype-specific (23 for Di-2, 5 for Ei-2, 14 for Ler-0, 15 for St-0, and 15 for Wt-1). The most commonly shared category among ecotypes was *translation* (GO:0006412; Di-2, Ler-0, St-2, and Wt-1), whereas a related category *RNA splicing via endonucleolytic cleavage and ligation* (GO:0000394), that is involved in tRNA processing, was down-regulated in three ecotypes Ei-2, Di-2 and Wt-1. Genes involved in *cell proliferation* (GO:0008283) were significantly down regulated in ecotypes L*er*-0, Di-2 and St-0. Down-regulated responses differentially elicited by Ei-2 includes processes of *killing of cells of other organism* (GO:0031640), shared with ecotype St-0, and a regulator of intracellular signaling and phosphate storage in plant seeds, *myo-inositol hexakisphosphate biosynthetic process* (GO:0010264), in common with Wt-1. *Ribosomal small subunit biogenesis* (GO:0042274), *protein maturation* (GO:0051604), *translational elongation* (GO:0006414), and *post-transcriptional regulation of gene expression* (GO:0010608) are all down-regulated categories common for Wt-1 and Di-2. Clearly these ecotypes had a lower protein expression as a part of infection response, whereas both ecotypes probably affected the same central processes. Negative regulation of protein biosynthesis referred to *protein folding* (GO:0006457), took also place in ecotypes L*er*-0 and Di-2. Besides, these ecotypes had down regulated the expression of genes belonging to categories *positive regulation of hydrolase activity* (GO:0051345) and *mitotic recombination* (GO:0006312). *Photorespiration* (GO:0009853) and *N-terminal protein myristoylation* (GO:0006499) were negatively affected in ecotypes St-0 and Di-2. Myristoylation can reversibly direct protein-protein and protein-lipid interactions and plays an essential role in membrane targeting and in a variety of signal transduction pathways^43^,^44^. Some viral proteins as in geminiviruses were also predicted to be modified with this kind of lipid anchor, this proteins localized into plasmodesmata and helps in cell-to-cell movement^45^. Down-regulation of *ethylene biosynthetic process* (GO:0009693) and *negative regulation of programmed cell death* (GO:0043069) have been seen for L*er*-0 and St-0, both categories probably allow the virus to evade the immune system. Functional categories *chromatin assembly or disassembly* (GO:0006333) and *microtubule-based process* (GO:0007017) were also down-regulated, suggesting that some cellular processes depending upon or altering of the microtubule cytoskeleton were impeded in L*er*-0 and Wt-1.

Some altered functions were common among ecotypes. These common functions are involved in the activation of immune defenses pathways such as the categories *jasmonic acid biosynthetic process, response to hydrogen peroxide, response to cyclopentenone* and to *cadmium ion*, and *detection of biotic stimulus*. Processes directly involved in TEV life cycle as *Golgi organization, vesicle-mediated transport, mRNA modification, plant-type cell wall modification*, and *defense response by callose deposition* were enriched in over-expressed genes. The biogenesis of basic plant resources was increased in plants infected with the evolved viruses, for example *negative regulation of catalytic activity, aspartate family amino acid catabolic process, photosystem II assembly, response to high light intensity*, and *secondary metabolite biosynthetic process* were all up-regulated. Considerable down-regulated responses have been seen for the protein biosynthesis and protein modification in host ecotypes L*er*-0, Ei-2 and Wt-1: *positive regulation of hydrolase activity, protein maturation, ribosomal small subunit biogenesis, protein folding, translational elongation, RNA splicing via endonucleolytic cleavage and ligation*, and *posttranscriptional regulation of gene expression and translation*. Cell proliferation was negatively affected by chromatin modifications and down-regulated mitotic recombination. Biosynthetic processes for ethylene, myoinositol hexakisphosphate and also photorespiration, needed for long time cell survival, were decreased in various ecotypes. Additionally, some functional categories of the immune response as negative regulation of programmed cell death or categories that could help in virus replication as *N-terminal protein myristoylation* and *microtubule-based process* were down-regulated. Despite a number of categories in common between pairs of ecotypes, there are many more categories that are solely observed in one ecotype (Tables 3 and 4). These ecotype-specific categories are covering a broad spectrum of functions, indicating that drivers of virus adaptation have been manifold and diverse in each ecotype. The perturbations induced in synthesis of basic cellular resources accompanied by symptoms development during virus infection was probably caused by interference of the virus in essential functions of the host, which may not be so important for the short-time survival of the plant.

Wrapping up the results from this section, although in all ecotypes at least one category was directly related to immune response, other functional groups were pervasively affected by the adaptation of the virus to its local host, indicating that general resistance mechanisms of the plant were not the main target for viral adaptation. In terms of changes of virus-host interactions caused by the adaptation of viral lineages to each host ecotype, they were frequent for lineages evolved in Ei-2, St-0 and Wt-1, as demonstrated by a larger number of differently affected genes in these ecotypes. Fewer of such evolved transcriptional modifications were found in L*er*-0 and Di-2. This division into two groups reflects the scheme of the similarities in the gene expression of ecotypes infected with ancestral virus and highlighted the importance of the host genotype for the evolutionary fate of TEV-*At*17b. The analysis of affected functional categories allows us to fathom what is happening in the hosts while the virus is adapting to them. Specific changes in the interactions, caused by different evolved lineages, had heterogeneous profiles among ecotypes, but also some intersections in affected functional categories were detected, indicating most redundant targets of viral adaptation. There were no intersections between all ecotypes. According to the similarities in functional responses, ecotypes could be classified into disjoint groups. It is worth mentioning that significant changes in functional up- and down-regulated categories were found between L*er*-0/2 and the ancestral virus in ecotype L*er*-0 despite no mutations were fixed in this lineage^32^, attributing the transcriptomic changes mostly to low-frequency subpopulations of viruses.

### Effect of evolution on new ecotypes in the interaction with the original ecotype L*er*-0

In a third set of analyses, we evaluated whether adaptation to each new host ecotype was associated with a change in the way evolved viruses interacted with the ancestral host ecotype L*er*-0. In other words, which changes in the way evolved viruses interact with the transcriptome of L*er*-0 plants may explain the negative pleiotropic fitness effects observed for some of the evolved lineages^32^. To do so, the transcriptomic profiles of L*er*-0 plants infected with each of the evolved viral lineages were contrasted to the transcriptome of L*er*-0 plants infected with the ancestral TEV-*At*17b (see “Methods: microarray data analyses” for a precise mathematical definition of the contrasts).

First, we identified the number of genes with altered expression in L*er*-0 plants infected with each of the evolved viruses compared with plants infected with the ancestral virus. A very minor number of significant alterations were found. Lineage Di-2/3 showed 1 under- and 2 over-expressed genes, lineage Ei-2/1 had 2 over- and 6 under-expressed genes, lineage L*er*-0/2 showed 1 over- and 1 under-expressed genes, lineage Wt-1/2 had 6 over-expressed genes, and lineage Wt-1/3 had 2 under- and 1 over-expressed genes. Two genes were shared by more than two lineages. The first gene was *PAP1* that encodes for a putative MYB DNA-binding domain containing transcription factor involved in anthocyanin metabolism and radical scavenging and is essential for the sucrose-mediated expression of the dihydroflavonol reductase gene. *PAP1* was also shown to be involved in symptoms development during *Tobacco mosaic virus* (TMV) infection^46^. The second gene, *At1g17147*, was shared between lineages Ei-2/1, L*er*-0/2 and Wt-1/3 and was under-expressed. No function has been proposed for this gene.

Other genes with altered expression on the different lineages are as follow. In lineage Di-2/3 over-expressed gene *At1g52000*, encoding for a mannose-binding lectin superfamily protein, is involved in response to salt stress. In Ei-2/1, under-expressed gene *At1g17345*, belonging to the small auxin up RNA (SAUR) like auxin-responsive protein family. Ei-2/1 also had 6 under-expressed genes, including *At3g05741* and *At1g09360*, both coding for proteins that belong to the superfamily of plant invertase/pectin methylesterase inhibitor, which is located in endomembrane system. Gene *PUP19,* coding for purine permease 19, is a member of a family of proteins related to *PUP1*, a purine transporter and is probably involved in the transport of purine and purine derivatives, across the plasma membrane. *At3g24065* and *At1g51230* are members of S1 family, plant self-incompatibility proteins located in the endomembrane system. In Wt-1/2 gene *PAP2*, another MYB domain containing protein whose main function is the production of anthocyanin pigment 2 and related to *PAP1*, was over-expressed. Another interesting over-expressed gene in Wt-1/2 was the apentatricopeptide repeat-containing protein (PPRP) encoded by gene *At5g12100*. The function of PPRPs in *A. thaliana* is currently not clear. However, it has been shown that a large number from this group of proteins interact with mitochondria and other organelles^47^ and that they are possibly involved in RNA editing^48^. Wt-1/3 over-expressed gene *At1g22490*, which encodes a basic helix-loop-helix (bHLH) DNA-binding superfamily protein, which is a sequence-specific DNA binding transcription factor, localized in nucleus. Wt-1/3 infection of L*er*-0 also resulted in down-regulation of gene *At3g15490* that encodes a regulatory protein for Vps4 activity in the multivesicular bodies’ pathway.

**Figure 4:**
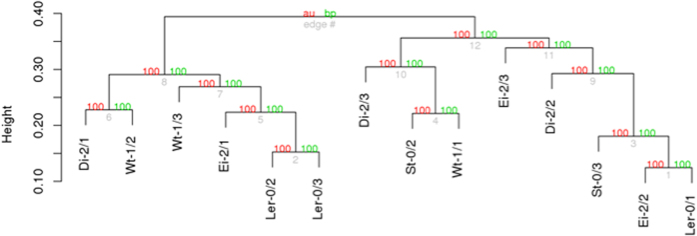
Similarity in transcriptomic profiles between plants of the L*er*-0 ecotype infected with each of the evolved viral lineages. Red numbers represent the approximately unbiased support of each cluster (percentage *P*-value) computed by multiscale bootstrapping. Green numbers represent the support of each cluster based on a standard bootstrapping. Grey numbers indicate the node label.

Next, we sought to evaluate how similar was the response of L*er*-0 to infection with the different evolved lineages. To do so, we computed Pearson correlation coefficients among the transcriptomic profiles obtained for each viral lineage when infecting L*er*-0. As before, the matrix of correlation coefficients was used to build a hierarchical cluster in which lineages cluster according to the similarities of the transcriptomic profiles of their L*er*-0 host (Fig. 4). In this case, all transcriptomes were significantly and positively correlated, with average similarities ranging between 0.736 (St-0 and Wt-1) and 0.899 (Ei-2 and L*er*-0). Clustering pattern shown in Fig. 4 reflects this high similarity in the response of Ler-0 to all the lineages: in sharp contrast to the clustering-by-local host shown in Fig. 1, now clustering does not reflects the local host ecotype in which viral lineages evolved.

Regarding differences in functional annotation, we have particularly address the following question: do some ecotypes select for viruses that induce smaller perturbations in the L*er*-0 transcriptome whereas others may select for viruses that induce larger perturbations? Table 5 summarizes the number of functional categories enriched in over- and under-expressed genes for each lineage. Counts data in Table 5 were fitted to a generalized linear model (GLM) with *ECOTYPE* and *LINEAGE* within *ECOTYPE* as factors and assuming a Poisson distribution for counts and a log-link function. This analysis shows that significant differences exist among replicates within ecotypes (likelihood ratio test *χ*^2^ = 12.215, 4 d.f., *P* = 0.016) and, more interestingly, among ecotypes (*χ*^2^ = 25.355, 9 d.f., *P* = 0.003), thus backing up the conclusion that lineages evolved in different hosts adapt to different factors, thus changing in slightly different manners the way they interact with the ancestral host L*er*-0. Furthermore, a Tukey’s *post hoc* test shows that the ranking of different ecotypes in the effect of their locally-evolved viral populations on L*er*-0 was St-0 > Wt-1 > L*er*-0 > Di-2 > Ei-2. Interestingly, this gradient of functional changes is negatively correlated with relative fitness in the unselected hosts and positively correlated with the virulence in the local host^32^: the most virulent lineages affected more functional categories when inoculated in L*er*-0, but had lower relative fitness in their new local ecotypes.

To summarize this section, the changes in virus-L*er*-0 interaction caused by subsequent evolution and adaptation to alternative ecotypes were small yet significant. The transcriptomic response was more homogeneous than when different host ecotypes were infected and compared, indicating that the genotype of the host is crucial in the virus-host interaction and that the cost of adaptation to new hosts is similar for all evolved lineages. The affected genes had diverse functions. Unfortunately, some are of unknown function and only one gene was in common in four lineages, *PAP1*. Analysis of functional transcriptomics were largely consistent with the analysis for viral fitness in L*er*-0^32^, as lineages evolved in ecotypes Ei-2 and Di-2 had affected the expression of less functional gene categories and also paid no fitness cost. St-0-evolved lineages showed an average fitness decrease of 8.9% in L*er*-0, being lineage St-0/3 the most specialized lineage; it also alters more functional categories than others. No homogeneous groups of evolved viral lineages could be defined according to the similarities in their perturbation of L*er*-0 functional profile, and not a clear clustering of lineages evolved in the same ecotype was found (Fig. 4).

### Evaluating differences in gene expression upon infection with generalist and specialist viruses

Finally, we were interested in answering the question of whether generalist and specialist viruses interact in a different manner with the different host ecotypes. Our hypothesis is that a specialist virus will show differences between the local and alternative hosts, whereas the generalist virus will interact similarly across all hosts. Therefore, we expected (1) that specialist and generalist viruses will alter the expression of different sets of host genes and that (2) generalist viruses will alter the expression of similar genes or functions across host ecotypes whereas specialist viruses will show a greater degree of heterogeneity. To test these predictions, we have characterized the transcriptome of plants from all ecotypes infected with the most generalist and the most specialist viruses evolved in our previous experiments^32^: lineage L*er*-0/1 was found to be a generalist virus, while lineage St-0/3 was qualified as the most specialist one^32^. In these cases, the statistical contrasts used were of the form “genes differentially expressed upon infection of foreign ecotypes with the generalist or specialist viruses” differ from “genes differentially expressed upon infection of the local ecotype with the generalist or specialist viruses” (see “Methods: microarray data analyses” for a precise mathematical definition of the contrasts).

**Figure 5:**
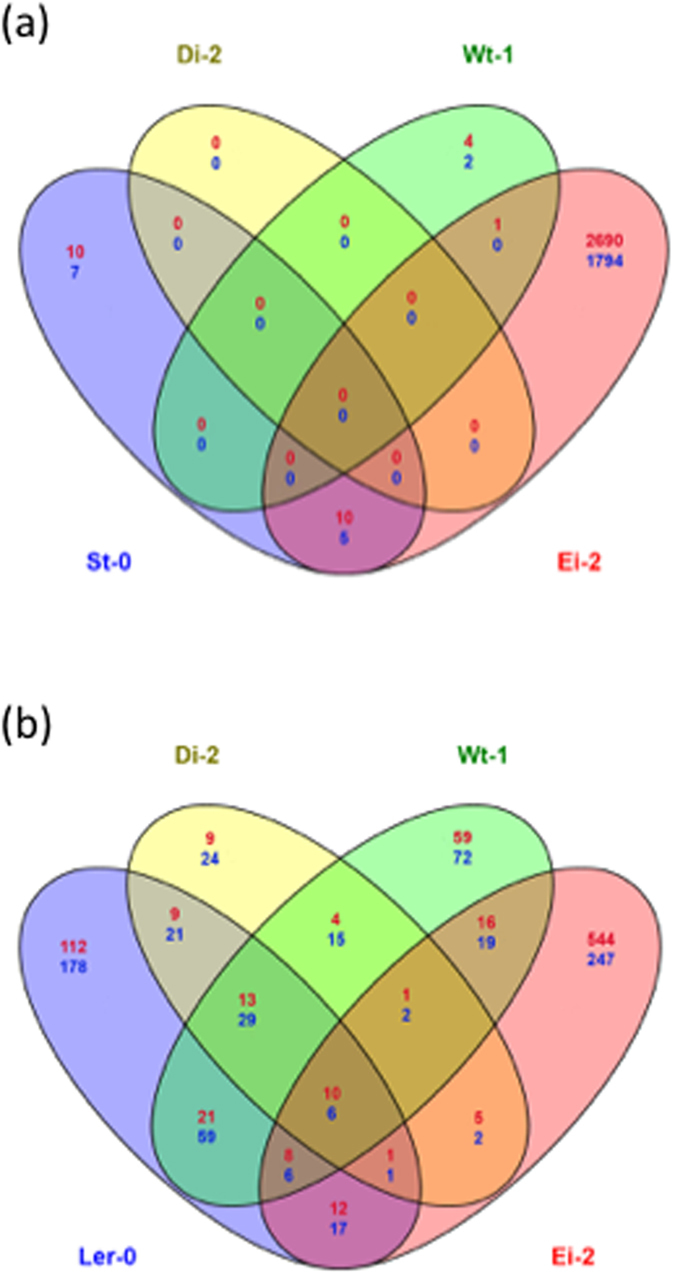
Venn diagrams illustrating the similarities in gene expression patterns across host ecotypes upon infection with (**a**) the most generalist virus lineage L*er*-0/1 and (**b**) the most specialist virus lineage St-0/3. In red, number of over-expressed genes; in blue number of under-expressed genes.

First, we evaluated gene expression differences between plants infected with both viruses. For the more generalist virus, L*er*-0/1, the expression values for infected plants from each ecotype were contrasted to the transcriptome of L*er*-0 plants (*i.e*., the local host for this viral lineage). In this way, the specific responses of ecotypes to the L*er*-0/1 virus could be compared. As shown in Fig. 5a, there are only a reduced number of differentially expressed genes when ecotypes are compared (median of 0, interquartile range 8), except in the case of the infection of ecotype Ei-2, where more than 3000 genes showed altered expression in an ecotype-specific manner. Following a similar logic, we contrasted the transcriptomic effect of infecting plants from each ecotype with St-0/3 to the transcriptomic effect of this virus on St-0 plants. Figure 5b shows that in this case the number of differentially expressed genes across ecotypes was more variable (median of 29.50, interquartile range 39) with, again Ei-2 showing the largest number of altered genes (791). Indeed, a signs test showed that both distributions of counts were significantly different (*P* = 0.002), with the generalist virus inducing a very similar perturbation across host ecotypes and the specialist virus inducing different sets of genes on each ecotype.

Transcriptomic responses of foreign ecotypes to infection with the St-0/3 specialist virus was different from the response of the local ecotype St-0. Ten over- and 6 under-expressed genes are in common for all ecotypes. Focusing first on the over-expressed genes, *At5g43060* encodes a granulin repeat cysteine protease-family protein involved in cell wall pectin metabolism and salt stress (TAIR database). *At4g34180* is also involved in salt stress and it belongs to the family of cyclase proteins, which localize in the plant cell wall but is of unknown function (TAIR database). Purine permease 18 (*PUP18*) is a member of a family of proteins related to purine transporter PUP1 and is probably involved in the transport of purine and purine-derivatives, such as cytokinins, across the plasma membrane^49^. *WRKY26* is DNA-binding protein 26, which regulates the activity of certain transcription factors and are involved in thermotolerance^50^. *XTH33* encodes a membrane-localized protein that is predicted to function during cell wall modification and could be one of the plant factors that condition the preference of aphids for symptomatic plants^51^. Over-expression of *XTH33* results in abnormal cell morphology^52^. *GLP5* encodes a plasmodesmata-located protein involved in regulating primary root growth by controlling phloem-mediated allocation of resources between the primary and lateral root meristems^53^, it is also involved in other processes such as response to stress^54^,^55^, response to acquired resistance, in ER-to Golgi mediated transport, and in amino acid transport. *At5g24490* encodes for a protein that is a constituent of the 30S ribosomal subunit and is involved in translation and in primary metabolic processes^56^,^56^. Senescence-associated gene *SEN1* is strongly induced by phosphate starvation^58^, and normally its transcripts are differentially regulated at the level of mRNA stability^59^; the senescence function of this gene has been associated with plant defense responses in *A. thaliana*^60^,^61^. Finally, serine carboxypeptidase-like 51 (*SCPL51*), a serine-type carboxypeptidase activity, is located in the endomembrane system and is involved in proteolysis^62^. Focusing in the 6 under-expressed genes that are in common for all ecotypes infected with the specialist virus St-0/3, *At2g44830* encodes for a member of the protein kinase superfamily involved in protein amino acid phosphorylation^63^. Zinc finger protein 5 (*ZFP5*) is involved in the regulation of trichome development^64^,^65^,^66^ and cytokinin signaling^67^. *At1g80620* is an RNA-binding protein and structural constituent of ribosome^68^,^69^. *PDR1* encodes for a multifunctional membrane protein with ATP-binding and ATPase activity, involved in biosynthetic processes of coumarin, flavonoid, lignin, and is also implicated in transmembrane transport and response to wounding^70^,^71^,^72^. *LHW* encodes a nuclear-localized transcriptional activator that promotes the production of stele cells in root meristems and is required to establish and maintain the normal vascular cell number and pattern in primary and lateral roots^73^,^74^. Finally, *At5g37970* encodes for a protein of the S-adenosyl-L-methionine-dependent methyltransferases superfamily whose biological function is unknown.

In summary, these results back up our original hypothesis: generalist virus L*er*-0/1 induces very similar perturbations in the transcriptomes of the different ecotypes analyzed. By contrast, the perturbations induced by the specialist virus St-0/3 are divergent among ecotypes.

**Figure 6:**
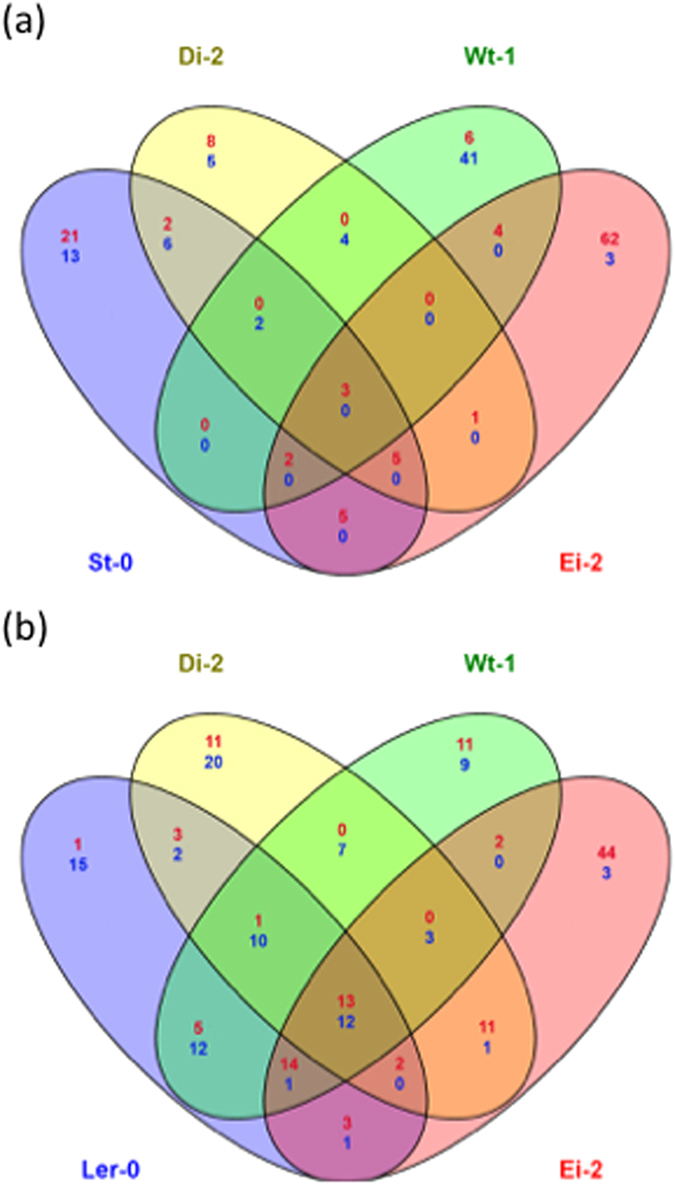
Venn diagrams illustrating the similarities in altered functional categories across host ecotypes upon infection with (**a**) the most generalist virus lineage L*er*-0/1 and (**b**) the most specialist virus lineage St-0/3. In red, number of up-regulated functional categories; in blue number of down-regulated functional categories.

In terms of biological functions, various enriched functional categories were found upon infection with both viral lineages, although a cluster analysis showed the functional responses were clearly different among viral isolates (data not shown). The number of significantly up- and down-regulated categories was similar between the specialist and generalist viral lineages. Nevertheless, the St-0/3 specialist has altered, in average, almost twice as many functional categories among ecotypes than the L*er*-0/1 generalist. The number of functional categories that are ecotype-specific or shared among ecotypes is summarized in Fig. 6a for plants infected with L*er*-0/1 and in Fig. 6b for plants infected with St-0/3. Three significantly up-regulated functional categories were common to all ecotypes when infected with the generalist virus L*er*-0/1 (Fig. 6a): *regulation of DNA replication* (GO:0006275), *cell proliferation* (GO:0008283), and *DNA replication initiation* (GO:0006270). No common down-regulated functional categories were shared by all five ecotypes infected with L*er*-0/1. The most common down-regulated functional categories included *protein import into nucleus* (GO:0006606), *response to cadmium ion* (GO:0046686), *translation* (GO:0006412), *detection of biotic stimulus* (GO:0009595), *salicylic acid biosynthetic process* (GO:0009697), *response to salt stress* (GO:0009651), *purine nucleotide biosynthetic process* (GO:0006164), and *negative regulation of defense response* (GO:0031348).

In the case of the infection with the specialist lineage St-0/3, there were disproportionally more affected categories in common between ecotypes than was observed for the generalist lineage, probably a consequence of the more even distribution of altered gene expressions induced by this viral isolate across ecotypes (Fig. 5). In total 13 functional categories were up-regulated by St-0 in all ecotypes, most of them largely implicated in immune responses. One of them is *respiratory burst involved in defense response* (GO:0002679) characterized by a phase of elevated metabolic activity, during which oxygen consumption increases as part of a defense response and leads, by an NADH dependent system, to the production of hydrogen peroxide, superoxide anions and hydroxyl radicals^75^,^76^. The second most commonly down-regulated functional category in plants infected with St-0/3 was the *response to chitin* (GO:0010200)^77^. Other functional categories directly related with viral infection include *protein targeting to vacuole* (GO:0006623), that is an important trait for maintenance of cell organization and function^78^, *growth regulator ethylene biosynthetic process* (GO:0009693) and *nucleosome assembly* (GO:0006334). Down-regulated were also functional categories for protein regulation as *response to misfolded protein* (GO:0051788), *proteasome core complex assembly* (GO:0080129) and *Golgi organization* (GO:0007030). Other altered functional categories, though maybe not directly related to viral infection, are *response to mechanical stimulus* (GO:0009612), *nitrate transport* (GO:0015706), *response to nitrate* (GO:0010167), *defense response to fungus* (GO:0050832), and *defense response to bacterium* (GO:0042742). Twelve up-regulated functional categories were found in common in ecotypes infected with the specialist lineage St-0/3. Two of them are related to the metabolism of nucleotides: *pyrimidine ribonucleotide biosynthetic process* (GO:0009220) and *RNA methylation* (GO:0001510), as a regulator of many diverse cellular processes. The largest group of common down-regulated functional categories is associated with tissue differentiation and plant growth, as for example *determination of bilateral symmetry* (GO:0009855), *meristem initiation* (GO: 0010014), thereby effecting growth and development of a plant by giving rise to more meristem or specialized tissue, *xylem and phloem pattern formation* (GO:0010051), *xylem development* (GO:0010089), *cell wall component xylan biosynthetic process* (GO:0045492), *glucuronoxylan metabolic process* (GO:0010413), *embryonic sac egg cell differentiation* (GO:0009560), *polarity specification of adaxial/abaxial axis* (GO:0009944), and *flower morphogenesis* (GO:0048439). Other down-regulated functional category was *protein targeting to mitochondrion* (GO:0006626), a process embedded into a functional network of several physiological processes.

Infection with the specialist lineage St-0/3 mainly activates defense mechanisms and down-regulates metabolic and plant growing processes in all foreign hosts. Specialist lineage St-0/3 induces functionally stronger and more diverse responses in foreign hosts. Many more differentially expressed genes were found in response to the infection of foreign hosts with the specialist virus than with the generalist virus, indicating that the gene expression profiles of the foreign hosts were clearly different from the local hosts in case of infection with the specialist lineage St-0/3, but not if infected with the generalist virus L*er*-0/1. However, these differences in gene expression vanished when functional categories were compared between specialist and generalist viruses. From this point of view, the transcriptomic response to infection with the specialist virus is more homogeneous. Ecotype Ei-2 shows an outstanding response profile if infected with L*er*-0/1 isolate, whose differential gene expression was thousand-fold higher than in other ecotypes. Nevertheless, as showed in functional profiling, the affected genes were involved in a similar number of biological functions than in other ecotypes, and there were even intersections in most categories. Analysis of enriched functional categories could identify changes in non-local hosts for both lineages. There were more common genes between non-local hosts infected with the specialist lineage St-0/3 than with the generalist lineage L*er*-0/1.

To summarize these functional analyses, we can say that the coordinated response of different plant ecotypes to viral infection was dependent on the infecting viral strain. Plant defense mechanisms were activated when the infecting virus was a specialist, and they were down-regulated when the infecting virus was a generalist. At the same time some metabolic process and plant growth were also down-regulated during infection with the specialist viral strain, but not with the generalist one. By contrast, cell proliferation and processes involved in DNA replication were up-regulated with the generalist strain but not with the specialist one.

## Conclusions

A large number of microarray RNA expression analyses have been done to investigate the evolution of the interaction between TEV-*At*17b and different ecotypes of its new host, *A. thaliana*, that differ in their susceptibility to infection with this virus. During a previous phase of experimental evolution, viral lineages evolved on each ecotype changed the way they interacted with its local and foreign hosts in an ecotype-specific manner. The most resistant ecotypes selected for more generalist viruses, able of infecting all ecotypes, whereas the most susceptible ecotypes selected for more specialist viruses. Transcriptional and functional profiling of plants of each ecotype infected with different evolved viral lineages have demonstrated that functionally more related ecotypes are more similar in their response to locally-adapted viruses, although heterogeneity in the response to virus infection among ecotypes was maintained. Transcriptional profiles of plants infected with the different viral lineages evolved on a common host ecotype were more similar among them than to the transcriptomic alterations induced by other viral lineages. These results suggest that host genetics plays a crucial role in the virus adaptation and that small intraspecific differences can determine the fate of virus evolution.

It is generally assumed that adaptation to a new host species comes with a cost in terms of fitness in the original host species^79^. However, this fitness cost was not evident when the host-range expansions occurs at the ecotype level; as we found no significant differences in fitness between the ancestral TEV-*At*17b and the evolved lineages when infecting the original host ecotype L*er*-0^32^. This lack of fitness differences is echoed at the transcriptomic level, since only a few small differences in expression profiles have been found between L*er*-0 plants infected with the ancestral TEV-*At*17b and the evolved viruses.

The evolved interaction between TEV-*At*17b derivatives and the five ecotypes of *A. thaliana* was not only based on the *RTM* locus but in the coordinated action of multiple genes that affect multiple biological functions. The most specialized viral lineage evolved in our experiments^32^ showed a more heterogeneous transcriptomic response across ecotypes than one of the most generalist lineages evolved. This observation suggests that a virus evolves as a generalist when natural selection favors the manipulation of similar sets of host genes across its host range. By contrast, a virus evolves as a specialist when natural selection favors fitness improvements by focusing in a subset of genes that are specific of the local host and do not interact efficiently with the transcriptomic of foreign hosts. Furthermore, differences between specialist and generalist viruses also exist in terms of functional categories. Infection with the most specialized viral isolate resulted in an activation of defense mechanisms and a down-regulation of functional categories involved in metabolic and plant growing processes. By contrast, infection with the most generalist isolate mainly altered very general and common targets of host transcriptome: up-regulation of processes involved in cell proliferation and DNA replication and down-regulation of host defenses and some specific biosynthetic processes.

## Methods

### Experimental evolution and cross-infection assay

The evolution experiment upon which this study was based is described in detail elsewhere^32^. In short, five *A. thaliana* ecotypes (Di-2, Ei-2, L*er*-0, St-0, and Wt-1) were inoculated with isolate TEV-*A*t17b, previously adapted to L*er*-0^19^,^30^. Three independent lineages were evolved per plant ecotype, resulting in 15 independent viral lineages. Twenty-one days post-inoculation (dpi), infection was confirmed by RT-PCR on upper leafs^29^, all systemically infected tissue collected, grounded into fine powder in liquid N_2_ and used to mechanically inoculate the next generation of plants from each ecotype as described elsewhere^19^,^29^,^30^,^31^,^32^. Experimental evolution was continued for 15 passages. After the evolution phase, all evolved viral lineages were inoculated on the five host ecotypes in a factorial design^32^. Fitness, virulence and infectivity was determined in all 15 × 5 = 75 combinations.

### Plant RNA extraction, RNA labeling and microarray hybridization

Four different microarray hybridization experiments were done: (1) every viral lineage infecting their corresponding local host, (2) all 15 viral lineages infecting the ancestral host L*er*-0, (3) the most specialist isolate St-0/3 infecting all five ecotypes, and (4) one of the most generalist isolates, Ler-0/1, also infecting all five ecotypes. In addition, we also have microarray data from the ancestral strain TEV-*At*17b infecting all five ecotypes^30^ (NCBI GEO accession GSE37269). Three biological replicates for each infected sample category and three replicates of mock-inoculated plants for each ecotype were processed. Four or five technical replicates of mock-inoculated plants were used for this study.

All plant inoculations performed with the 15 evolved TEV lineages were done in a single experimental block. After inoculation, plants were maintained in a BSL-2 greenhouse at 16:8 h light:dark and 24:20 °C day:night until sample collection 21 dpi. Symptomatic plants were collected, the whole plants (excluding roots) were ground into fine powder and stored at −80 °C. In this regard, the transcriptomes will represent a snapshot, of an otherwise very dynamic process, taken at a time which is only relevant in the context of the experimental evolution described above: the stage of infection at which viral populations were sampled to perform the next evolution passage. Since all plant tissues were pooled and homogenized together, the transcriptomes will also represent an average from different cell types and tissues.

Total RNA was extracted from these homogenized tissue of control and infected plants using the RNeasy Plant Mini kit (Qiagen) and following manufacturer’s protocol. RNA integrity was verified in an Agilent 2100 Bioanalyzer (Agilent Technologies). RNA samples for Bioanalyzer were prepared using RNA 6000 Nano Assay Kit (Agilent Technologies) following manufacturer’s instructions. Five hundred nanograms of spectrophotometrically quantified RNA (Nanodrop ND1000, Thermo Scientific) were used in amplification and labeling reaction with the Quick Amp Labeling Kit One-Color (Agilent Technologies) following manufacturer’s instructions. All samples were amplified and labeled with Cy3 and subsequently verified in a 2100 Bioanalyzer as previously described. As positive control of amplifying, labeling, and hybridization, synthetic RNA Spikes were added to the samples. The spike solution was diluted and prepared following manufacturer’s protocol (RNA Spike-In Kit, One-Color, Agilent Technologies). Labeled RNA was used to hybridize the microarray 4 × 44 K slides carrying *A. thaliana* Col-0 probes (Agilent Technologies) as described at the standard hybridization protocol from Gene Expression Hybridization Kit (Agilent Technologies). After hybridization and wash, slides were scanned at 532 nm with a GenePix 4000B scanner (Axon Molecular Devices), at 10 μm resolution and 100% laser power. Photomultiplier tube voltages were adjusted to equal the overall signal intensity for each channel, to increase signal-to-noise ratio, and to reduce the number of spots with saturated pixels. Spot intensities were quantified using GenePix Pro 4.1 software (Axon Molecular Devices).

### Microarray data analyses

Agilent ProcessedSignal (Agilent Feature Extraction Software) was standardized using quantile normalization^80^. Differential gene expression was carried out using the Limma package from Bioconductor^80^,^81^. Multiple testing adjustment of *P*-values was done according to Benjamini and Hochberg methodology^82^. Genes were considered significantly over- or under-expressed when having an adjusted *P*-value (false discovery rate, FDR) less than 0.05.

In the first set of analyses, we were interested in identifying genes differentially expressed in infected versus mock-inoculated control plants. The corresponding contrasts were of the form 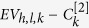, where *EV*_*h,l,k*_ is the transcriptomic profile generated from a plant of genotype *k* ∈ {Di-2, Ei-2, L*er*-0, St-0, Wt-1} when it is infected with lineage *l* ∈ {1,2,3} of a virus evolved in local host *h* ∈ {Di-2, Ei-2, L*er*-0, St-0, Wt-1} and 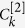 is the transcriptomic profile generated from a control plant of the same genotype *k*. Superscript 2 refers to the controls generated for this work. A total of 15 of such contrasts were evaluated. In a second set of analyses, we were interested in evaluating which transcriptomic changes were ecotype-specific versus general. In this case contrasts took the form 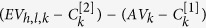, where *AV*_*k*_ is the transcriptomic profile generated from a plant of genotype *k* infected with the ancestral TEV-*At*17b, and 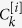 is the transcriptomic profile generated from a control plant of the same genotype *k*, and superscript *i* refers either to the controls generated in ref. (30) (*i* = 1) or specifically for this work (*i* = 2). A total of 15 of such contrasts were evaluated. In a third set of analyses, we were interesting in analyzing the cost of adaptation to new host ecotypes in terms of performance in the original host ecotype L*er*-0. Contrasts were now of the form 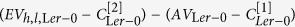, where 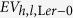 is the transcriptomic profile generated from a plant of genotype L*er*-0 when it is infected with lineage *l* ∈ {1, 2, 3} of a virus evolved in local host *h* ∈ {Di-2, Ei-2, L*er*-0, St-0, Wt-1}, 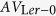 is the transcriptomic profile generated from a plant of genotype L*er*-0 infected with the ancestral TEV-*At*17b, and 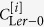 is the transcriptomic profile generated from a control plant of genotype L*er*-0, Superscript *i* ∈ {1, 2} as described above. A total of 15 of such contrasts were evaluated. Finally, we evaluated the differences in terms of transcriptomic alterations induced by specialist and generalist viruses. Contrasts for this study were of two specific forms. First, 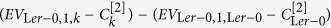, where 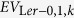 is the transcriptomic profile generated from a plant of genotype *k* ∈ {Di-2, Ei-2, St-0, Wt-1} when it is infected with generalist lineage L*er*-0/1. Second, 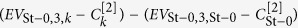, where 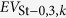 is the transcriptomic profile generated from a plant of genotype *k* ∈ {Di-2, Ei-2, L*er*-0, Wt-1} when it is infected with specialist lineage St-0/3. A total of four of each class of contrasts were evaluated.

Gene set analysis (GSA) was carried out for identifying significant GO terms using a logistic regression model (LRpath)^33^,^34^. This method detects significantly up- or down-regulated blocks of functionally related genes in lists of genes ordered by differential expression. Given that many functional terms were simultaneously tested; the results of the test were corrected for multiple testing with the FDR^82^,^83^ method to obtain an adjusted *P*-value. GO annotation for the genes in the microarray where taken from ENSEMBL 73 release (http://www.ensembl.org).

Clustering analyses were used to classify transcriptomic responses and functional profiles according to their similarities. The agglomerative method used in hierarchical clustering was UPGMA (unweighted pair group method with arithmetic mean)^84^,^85^. This particular hierarchical clustering method starts by calculating the all-to-all distance matrix. The two closest patterns are merged and the all-against-all distance matrix is calculated again but using the new cluster instead of the two merged patterns. This process is repeated until the complete dendrogram is built. Pearson product-moment correlation coefficients between pairs of transcriptomic or functional profiles were used as a measure of their similarity. When comparing two expression profiles, a significant correlation may indicate that those few genes that change, are *exactly the same* in both samples, showing a similar expression pattern. If this is the case, the correlation coefficient is expected to be high. If there are few genes with differential expression but they do not match in the two samples being compared, then the correlation will be lower. This is independent on whether or not the distribution of gene expression is Gaussian. When comparing two transcriptomes, correlations were computed among the values of the corresponding contrast statistics obtained for each probe set in the microarray. When comparing two GO functional profiles, correlations were computed among the odds ratio obtained for each biological process evaluated. The statistical support of each cluster was evaluated using two techniques: (1) approximately unbiased support of each cluster (percentage *P*-value) computed by multiscale bootstrapping and (2) standard bootstrapping^86^.

All microarray data generated in this study were deposited at NCBI GEO under accession GSE66020.

## Additional Information

**How to cite this article**: Hillung, J. *et al*. The transcriptomics of an experimentally evolved plant-virus interaction. *Sci. Rep.* **6**, 24901; doi: 10.1038/srep24901 (2016).

## Figures and Tables

**Table 1:** Similarity in transcriptional profiles among infected ecotypes.

	Di-2	Ei-2	L*er*-0	St-0	Wt-1
Di-2	0.615 ± 0.004	−0.074 ± 0.005	0.407 ± 0.005	−0.210 ± 0.005	0.021 ± 0.006
Ei-2		0.857 ± 0.002	0.036 ± 0.005	0.468 ± 0.005	0.584 ± 0.004
L*er*-0			0.647 ± 0.004	−0.185 ± 0.005	0.260 ± 0.005
St-0				0.784 ± 0.003	0.359 ± 0.005
Wt-1					0.842 ± 0.003

Similarities are measured using Pearson correlation coefficient (±1 SEM). Correlations were computed among the values of the corresponding contrast statistics obtained for each probe set in the microarray (see Methods: microarray data analyses section for details). All cases are statistically significant (*P* < 0.001).

**Table 2:** Similarity in functional profiles (lists of enriched GO functions) among infected ecotypes.

	Di-2	Ei-2	L*er*-0	St-0	Wt-1
Di-2	0.709 ± 0.019	0.279 ± 0.026	0.441 ± 0.025	−0.131 ± 0.027	0.250 ± 0.027
Ei-2		0.919 ± 0.011	0.310 ± 0.026	0.136 ± 0.027	0.667 ± 0.021
L*er*-0			0.833 ± 0.015	−0.283 ± 0.026	0.581 ± 0.022
St-0				0.721 ± 0.019	−0.001 ± 0.028
Wt-1					0.793 ± 0.016

The degree of similarity was measured using Pearson correlation coefficient (±1 SEM). Correlations were computed among the odds ratio obtained for each biological process being evaluated (see Methods: microarray data analyses section for details). All cases are statistically significant (*P* < 0.001).

**Table 3:** Up-regulated functional clusters of GO terms for biological processes.

Functional clusters	Di-2	Ei-2	L*er*-0	St-0	Wt-1
chitin catabolic process	✓				
Golgi organization	✓	✓			
abscission	✓				
response to far red light	✓				
cation homeostasis	✓		✓		
iron ion transport	✓				
maltose metabolic process	✓		✓		
nitrate assimilation	✓				
anthocyanin-containing compound biosynthesis	✓		✓		
response to wounding	✓	✓			✓
negative regulation of catalytic activity	✓			✓	
response to nitrate	✓				
myo-inositol hexakisphosphate biosynthetic process	✓				
cellular response to iron ion starvation	✓				
photosystem II assembly	✓			✓	
para-aminobenzoic acid metabolic process	✓		✓		
drug transmembrane transport	✓	✓			
cell proliferation		✓			
vesicle-mediated transport		✓			✓
positive regulation of catalytic activity		✓			
cell wall organization or biogenesis		✓			
response to karrikin		✓			
plant-type cell wall biogenesis		✓	✓		
protein folding		✓		✓	✓
acetyl-CoA metabolic process		✓			
multidimensional cell growth		✓			
anther development		✓			
ethylene biosynthetic process		✓			✓
vitamin metabolic process		✓			
cytokinesis by cell plate formation		✓			
mRNA modification		✓		✓	
cellulose metabolic process		✓			
toxin catabolic process		✓			
detection of biotic stimulus		✓			✓
cell surface receptor signaling pathway		✓			
DNA replication initiation		✓			
response to cyclopentenone		✓			✓
cellular homeostasis		✓			
dicarboxylic acid biosynthetic process		✓			
ncRNA metabolic process		✓			
carotenoid biosynthetic process		✓			
regulation of protein dephosphorylation		✓			
monocarboxylic acid transport		✓			
defense response by callose deposition		✓			✓
ribonucleoside monophosphate biosynthetic process		✓			
organelle localization		✓			
response to cadmium ion		✓	✓		
nucleoside transport		✓			
aspartate family amino acid catabolic process		✓			✓
proteolysis			✓		
water transport			✓		
vacuole organization			✓		
response to symbiotic fungus			✓		
tricarboxylic acid cycle			✓		
lipid modification			✓		
macromolecule catabolic process			✓		
response to hexose			✓		
response to salt stress			✓		
pollen exine formation			✓		
divalent metal ion transport			✓		
glucosinolate biosynthetic process			✓		
response to high light intensity				✓	✓
xylem development				✓	
photosynthesis				✓	
actin filament-based movement				✓	
hydrogen peroxide biosynthetic process				✓	
protein desumoylation				✓	
secondary metabolic process				✓	
mRNA export from nucleus				✓	
polysaccharide catabolic process				✓	
mRNA metabolic process				✓	
cofactor biosynthetic process				✓	
secondary metabolite biosynthetic process				✓	✓
alcohol metabolic process				✓	
killing of cells of other organism				✓	
response to hydrogen peroxide				✓	✓
regulation of cellular component organization				✓	
determination of bilateral symmetry				✓	
cellular response to phosphate starvation				✓	
positive regulation of transcription, DNA-templated				✓	
transmembrane transport				✓	
plant-type cell wall modification				✓	✓
jasmonic acid biosynthetic process				✓	✓
pyrimidine ribonucleotide biosynthetic process				✓	
seed germination				✓	
regulation of developmental growth				✓	
RNA 3'-end processing					✓
respiratory electron transport chain					✓
pectin catabolic process					✓
recognition of pollen					✓
negative regulation of programmed cell death					✓
signal peptide processing					✓
N-terminal protein myristoylation					✓
phosphate ion transport					✓
endocytosis					✓
oligopeptide transport					✓
galactolipid biosynthetic process					✓
glycerol metabolic process					✓

Left column represent super categories of GO terms. The rest of columns indicate whether the super category was up-regulated in the corresponding ecotype.

**Table 4:** Down-regulated functional clusters of GO terms for biological processes.

Functional clusters	Di-2	Ei-2	L*er*-0	St-0	Wt-0
acetyl-CoA metabolic process	✓				
circadian rhythm	✓				
cell proliferation	✓		✓	✓	
tissue development	✓				
response to ionizing radiation	✓				
ribosomal small subunit biogenesis	✓				✓
positive regulation of hydrolase activity	✓		✓		
protein maturation	✓				✓
photorespiration	✓			✓	
transcription factor import into nucleus	✓				
cell-cell signaling	✓				
mitotic recombination	✓		✓		
microtubule-based movement	✓				
mitotic cell cycle	✓				
dicarboxylic acid biosynthetic process	✓				
response to molecule of bacterial origin	✓				
regulation of cell proliferation	✓				
response to endoplasmic reticulum stress	✓				
cell redox homeostasis	✓				
mRNA metabolic process	✓				
protein folding	✓		✓		
nucleus organization	✓				
cullin deneddylation	✓				
pseudouridine synthesis	✓				
DNA-templated transcription, elongation	✓				
regulation of nucleoside metabolic process	✓				
translational elongation	✓				✓
proteasomal protein catabolic process	✓				
RNA endonucleolytic cleavage and ligation	✓	✓			✓
posttranscriptional regulation of gene expression	✓				✓
glycerophospholipid biosynthetic process	✓				
peptidyl-amino acid modification	✓				
N-terminal protein myristoylation	✓			✓	
translation	✓		✓	✓	✓
vernalization response	✓				
killing of cells of other organism		✓		✓	
protein import into peroxisome matrix		✓			
abscission		✓			
myo-inositol hexakisphosphate biosynthetic process		✓			✓
chlorophyll catabolic process		✓			
gibberellic acid mediated signaling pathway		✓			
seed dormancy process		✓			
ethylene biosynthetic process			✓	✓	
cytoplasmic transport			✓		
response to decreased oxygen levels			✓		
chromatin assembly or disassembly			✓		✓
anther development			✓		
microtubule-based process			✓		✓
actin filament-based movement			✓		
cytokinesis by cell plate formation			✓		
toxin catabolic process			✓		
salicylic acid biosynthetic process			✓		
response to cyclopentenone			✓		
negative regulation of programmed cell death			✓	✓	
RNA methylation			✓		
ER-nucleus signaling pathway			✓		
cell fate specification			✓		
response to hydrogen peroxide			✓		
pyrimidine ribonucleotide biosynthetic process			✓		
heat acclimation			✓		
detection of biotic stimulus				✓	
proteasome core complex assembly				✓	
regulation of G2/M transition of mitotic cell cycle				✓	
ER to Golgi vesicle-mediated transport				✓	
response to mechanical stimulus				✓	
auxin biosynthetic process				✓	
response to glucose				✓	
respiratory burst involved in defense response				✓	
regulation of hydrogen peroxide metabolic process				✓	
DNA replication initiation				✓	
MAPK cascade				✓	
positive regulation of flavonoid biosynthesis				✓	
plant-type cell wall cellulose metabolic process				✓	
ubiquitin-dependent protein catabolic process				✓	
cellular response to hypoxia				✓	
response to gibberellin					✓
vegetative phase change					✓
protein targeting to chloroplast					✓
DNA recombination					✓
maltose metabolic process					✓
photosynthetic electron transport in photosystem I					✓
cell cycle process					✓
response to gamma radiation					✓
protein deubiquitination					✓
nuclear-transcribed mRNA catabolic process					✓
auxin polar transport					✓
polarity specification of adaxial/abaxial axis					✓
regulation of protein modification process					✓
leaf morphogenesis					✓
regulation of gene expression, epigenetic					✓

Left column represent super categories of GO terms. The rest of columns indicate whether the super category was down-regulated in the corresponding ecotype.

**Table 5:** Characterization of the response of L*er*-0 ecotype to infection with all the evolved viral lineages.

Local Host	Lineage	Down-regulated	Non-altered	Up-regulated
Di-2	1	28	1269	23
	2	109	1138	73
	3	97	1091	132
Ei-2	1	65	1236	19
	2	81	1137	102
	3	124	1192	4
L*er*-0	1	135	1133	52
	2	112	1195	13
	3	129	1159	32
St-0	2	120	1128	72
	3	131	1163	26
Wt-1	1	40	1264	16
	2	316	1004	0
	3	96	1198	26

Count of functional classes enriched in each comparison with L*er*-0 plants infected with the ancestral TEV-*At*17b virus.
